# Expert consensus on the diagnosis and therapy of endo-periodontal lesions

**DOI:** 10.1038/s41368-024-00320-0

**Published:** 2024-09-01

**Authors:** Bin Chen, Yanan Zhu, Minkui Lin, Yangheng Zhang, Yanfen Li, Xiangying Ouyang, Song Ge, Jiang Lin, Yaping Pan, Yan Xu, Yi Ding, Shaohua Ge, Faming Chen, Zhongchen Song, Shaoyun Jiang, Jiang Sun, Lijun Luo, Junqi Ling, Zhi Chen, Lin Yue, Xuedong Zhou, Fuhua Yan

**Affiliations:** 1 Department of Periodontology, Nanjing Stomatological Hospital, Affiliated Hospital of Medical School, Institute of Stomatology, Nanjing University, Nanjing, China; 2 Department of Endodontics, Nanjing Stomatological Hospital, Affiliated Hospital of Medical School, Institute of Stomatology, Nanjing University, Nanjing, China; 3 Clinical Research Center for Oral Tissue Deficiency Diseases of Fujian Province & Fujian Key Laboratory of Oral Diseases & Fujian Provincial Engineering Research Center of Oral Biomaterial, School and Hospital of Stomatology, Fujian Medical University, Fuzhou, China; 4 Department of Periodontology, Peking University School and Hospital of Stomatology & National Center for Stomatology & National Clinical Research Center for Oral Diseases & National Engineering Research Center of Oral Biomaterials and Digital Medical Devices, Beijing, China; 5 School and Hospital of Stomatology, Zunyi Medical University, Zunyi, China; 6 Department of Stomatology, Beijing TongRen Hospital, Capital Medical University, Beijing, China; 7 Department of Periodontics, School and Hospital of Stomatology, China Medical University, Shenyang, China; 8 Department of Periodontology, The Affiliated Stomatological Hospital of Nanjing Medical University, Jiangsu Key Laboratory of Oral Diseases, Nanjing Medical University, Nanjing, China; 9 State Key Laboratory of Oral Diseases & National Center for Stomatology & National Clinical Research Center for Oral Diseases & Department of Periodontics, West China Hospital of Stomatology, Sichuan University, Chengdu, China; 10 Department of Periodontology, School and Hospital of Stomatology, Shandong University & Shandong Key Laboratory of Oral Tissue Regeneration & Shandong Engineering Research Center of Dental Materials and Oral Tissue Regeneration & Shandong Provincial Clinical Research Center for Oral Diseases Jinan, Jinan, China; 11 State Key Laboratory of Oral & Maxillofacial Reconstruction and Regeneration, National Clinical Research Center for Oral Diseases, Shaanxi International Joint Research Center for Oral Diseases, Department of periodontology, School of Stomatology, The Fourth Military Medical University, Xi’an, China; 12 Department of Periodontology, Shanghai Ninth People’s Hospital, Shanghai Jiao Tong University School of Medicine; College of Stomatology, Shanghai Jiao Tong University; National Center for Stomatology; National Clinical Research Center for Oral Diseases; Shanghai Key Laboratory of Stomatology; Shanghai Research Institute of Stomatology, Shanghai, China; 13 Department of Periodontology, Stomatological Center, Peking University Shenzhen Hospital, Shenzhen, China; 14 Dalian Stomatological Hospital, Dalian, China; 15 Department of Periodontology, Stomatological Hospital and Dental School of Tongji University, Shanghai Engineering Research Center of Tooth Restoration and Regeneration, Shanghai, China; 16 Department of Operative Dentistry and Endodontics, the Affiliated Stomatological Hospital of the Guanghua School of Stomatology, Sun Yat-sen University, Guangzhou, China; 17 The State Key Laboratory Breeding Base of Basic Science of Stomatology (Hubei-MOST) & Key Laboratory of Oral Biomedicine Ministry of Education, School and Hospital of Stomatology, Wuhan University, Wuhan, China; 18 Department of Cariology and Endodontology, Peking University School and Hospital of Stomatology & National Clinical Research Center for Oral Diseases & National Engineering Research of Oral Biomaterials and Digital Medical Devices & Beijing Key Laboratory of Digital Stomatology, Peking University, Beijing, China; 19 State Key Laboratory of Oral Diseases & National Center for Stomatology & National Clinical Research Center for Oral Diseases & Department of Cariology and Endodontics, West China Hospital of Stomatology, Sichuan University, Chengdu, China

**Keywords:** Periodontitis, Oral diseases

## Abstract

Endo-periodontal lesions (EPLs) involve both the periodontium and pulp tissue and have complicated etiologies and pathogenic mechanisms, including unique anatomical and microbiological characteristics and multiple contributing factors. This etiological complexity leads to difficulties in determining patient prognosis, posing great challenges in clinical practice. Furthermore, EPL-affected teeth require multidisciplinary therapy, including periodontal therapy, endodontic therapy and others, but there is still much debate about the appropriate timing of periodontal therapy and root canal therapy. By compiling the most recent findings on the etiology, pathogenesis, clinical characteristics, diagnosis, therapy, and prognosis of EPL-affected teeth, this consensus sought to support clinicians in making the best possible treatment decisions based on both biological and clinical evidence.

## Introduction

Endo-periodontal lesions (EPLs), formerly known as retrograde periodontitis, endodontic-periodontal lesions, periodontal-endodontic lesions, or periodontic-endodontic lesions, affect the periodontium as well as the dental pulp and can interact with one another through pathological communication pathways^1–5^. Due to the multiple etiologies of EPLs, the clinical presentations and classifications are diverse, and the corresponding therapies are different.

Our understanding of the pathophysiology, pathogenesis, and other aspects of EPLs has continued to improve over time^2,4–6^. However, determining the optimal therapy for EPLs remains very challenging. In 2018, the World Workshop on the Classification of Periodontal and Peri-implant Disease and Conditions proposed a new classification system based on the prognosis of the involved tooth^4^, which aids in the treatment decision-making process. This manuscript summarized the progress in elucidating the etiology, pathogenesis, clinical features, diagnosis, therapy, and prognosis of EPLs to help clinicians select an optimal therapy based on both biological and clinical evidence.

## Etiology and pathogenesis

The pulp and periodontal tissues are anatomically connected, allowing infections and lesions affecting one tissue to the other tissue, leading to the occurrence of combined lesions. The pathogenesis of EPLs is multifactorial and involves major risk factors such as microbiological elements, pathological communication routes, trauma, and iatrogenic diseases^7–11^.

### Anatomical relationships between periodontal and pulp tissues

**Fig. 1:**
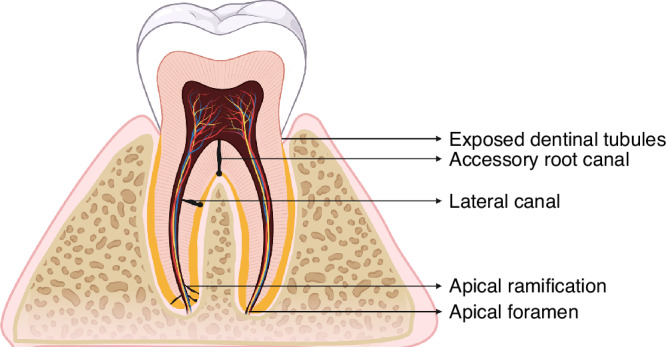
Anatomical relationships between periodontal and pulp tissues. (Created with BioRender.com)

Dental pulp and the periodontium are closely connected through the apical foramen, lateral root canals and accessory canals, and the dentinal tubules communicate with the pulp and periodontal tissues, forming three main avenues for communication (Fig. 1), which are the anatomical basis of EPLs^1^. When infection occurs, pathogens and their products migrate through these transit channels between dental pulp and periodontal tissues^2,7^.

1. **Apical foramen**: As a physiological anatomical structure, the apical foramen is the principal and most direct route of communication between the dental pulp and periodontium surrounding the root apex. Once the dental root pulp is infected, pathogens and their byproducts can easily reach to the periapical area through the apical foramen, which causes local inflammatory vascular reactions, promotes the absorption of alveolar bone, cementum, and dentine; destroys the integrity of periodontal tissues; and eventually leads to EPL formation^1,12^. In contrast, when periodontal damage reaches the apical area, periodontal infection can also cause pulp infection in a retrograde direction through the apical foramen^13^.
2. **Lateral and accessory canals**: The lateral and accessory canals, including the intercanal anastomose, lateral canal, accessory root canal, apical furcation, and apical ramification, are another route of infective communication in EPLs. It is estimated that 30–40% of all teeth have lateral or accessory canals, the majority of which are found in the apical third of the root (17.0%). These canals are less frequently found in the middle third (approximatedly 9%) and least frequently in the coronal third (less than 2%)^14,15^. Due to their small structure and irregular distribution, lateral root canals are blind areas in which completely removing infected materials and tightly sealing these canals are difficult during root canal therapy are difficult, thus becoming a source of secondary periodontal infection^16–18^.
3. **Exposed dentinal tubules:** Exposed dentinal tubules may serve as viable communication pathways between dental pulp and periodontium^1^. The cementum is so thin (16–50 μm) that it is easily removed by inappropriate tooth brushing or treatment. Importantly, the same tooth may have different cementoenamel junction characteristics, resulting in dentin exposure on one surface with complete cementum coverage on other surfaces^19^. EPLs caused by dentin tubule exposure should be considered in case of pulp infection without a clear source of hard tissue injury or after scaling and root planning (SRP), trauma or bleaching^20,21^.

### Microbiology

EPLs have a microbial etiology^1,9,10^. Recently, the application of metagenomic next-generation sequencing and progress in oral microecology theory have broadened the microbial etiologies of EPLs^22^. Fungi, archaea and viruses have been found to be associated with pulpitis, apical periodontitis and periodontitis. However, bacteria are by far the most prevalent and dominant microorganisms in endodontic or periodontal infections^23^.

Based on the commonalities in anaerobic microecology between the root canal system and the periodontal pocket, anaerobic microorganisms have adapted to grow simultaneously. There is substantial similarity and species crossover between the microbiota found in infected root canals and periodontal pockets^22^. Periodontal pathogen groups called “red and orange complexes”^24^, such as *Porphyromonas gingivalis*, *Tannerella forsythia*, *Fusobacterium*, *Prevotella*, and *Treponema denticola*, have also been detected in infected root canal systems^25–27^. In addition, the most prevalent and abundant bacterial species/phylotypes in endodontic infections, including *Fusobacterium nucleatum, Dialister species, Porphyromonas endodontalis, Porphyromonas gingivalis, Prevotella species, Tannerella forsythia, Treponema species*, *Pseudoramibacter alactolyticus, Actinomyces species and Streptococcus species*)^28^, have also been found in periodontal pockets.

### Other facilitating factors

Some unique conditions, such as developmental malformations, tooth fractures or cracks, root resorption, and iatrogenic factors, may also form communication pathways between the dental pulp and the periodontium, resulting in EPLs^1,7,29^.

1. **Developmental malformations:** Abnormal tooth structure and tooth morphology, such as lingual fossa malformation, root groove malformation, and lingual cusp malformation, may be precipitating factors of EPLs^29,30^.
2. **Tooth fractures or cracks:** Tooth fractures or tooth cracks, which are usually caused by traumatic injuries or occlusal trauma, can result in the formation of pathological communication pathways between the periodontium and pulp, leading to EPLs.
3. **Root resorption:** Root resorption is multifactorial, and the most important etiological factors are trauma, pulp infection, tooth bleaching, and orthodontic treatment. In some cases, root resorption without clear external causes is thought to be related to age, race, occlusal characteristics, and genetic susceptibility^31^.
4. **Iatrogenic factors:** Iatrogenic factors, such as imperfect root canal treatment, defective prosthetics, inappropriate periodontal scaling, and perforation of the lateral wall of the root canal or the pulp chamber floor, may increase the chance of communication between periodontium and dental pulp, resulting in EPL formation^32^.

- Inadequate root canal therapy is an important risk factor for EPLs, including incomplete removal of infected substances from the root canal system, and omission of the root canal or untreated collateral canal, is an important risk factor for EPLs. An infection from the root canal can then spread to periodontal tissues and result in EPLs^33,34^.
- Inadequate coronal sealing of the teeth after root canal treatment can lead to coronal microleakage and leakage along the root canal^35^. Although the conclusions vary, all the literature supports the importance of adequate coronal restoration on periapical status^36,37^.
- Perforations of the lateral wall of the root canal or the floor of the pulp chamber are usually caused by extensive caries, internal root resorption, and operation errors in preparing the canal post during root canal treatment or repair, and the resulting infection migrates and progresses rapidly, leading to a negative prognosis^38^.

## Classification

### Previous classification

In 1972, Simon et al. published the first EPL classification system, which had been widely used for decades, including the following five main categories: primary endodontic lesions; primary endodontic lesions with secondary periodontal involvement; primary periodontal lesions; primary periodontal lesions with secondary endodontic involvement and “true” combined lesions^6^. In 1996, Torabinejad et al.^39^ proposed a revised EPL classification system based on the origin of the periodontal pocket: endodontic origin; periodontal origin; combined endodontic-periodontal lesions; separate endodontic and periodontal lesions; lesions with communication and lesions with no communication. In 1999, the American Society of Periodontology first incorporated EPLs into the Classification System of Periodontal Disease and named it “combined periodontic-endodontic lesions”, which was of limited utility due to the lack of categories^40^.

In 2014, Al-Fouzan et al.^41^ proposed a new endodontic-periodontal interrelationship classification system based on the primary disease and its secondary effect, as follows: retrograde periodontal disease, including primary endodontic lesions with drainage through the periodontal ligament, and primary endodontic lesions with secondary periodontal involvement; primary periodontal lesions; primary periodontal lesions with secondary endodontic involvement; combined endodontic-periodontal lesions and iatrogenic periodontal lesions.

The above classification is based on the assumption that the prognosis of EPL of periodontal origin is worse than that of pulp-derived lesion. However, it is not feasible to use “medical history” as the primary criterion for diagnosis, as it is difficult to determine whether the lesion is from an endodontic source, periodontal origin, or a combined endodontic-periodontal lesion^7^. In addition, identifying the primary source of infection of the lesion may not be relevant to the therapy of EPLs, as both of root canal and periodontal therapy are essential for EPLs involving both the periodontium and pulp^42^.

### New classification of periodontal and peri-implant diseases and conditions in 2018

**Fig. 2:**
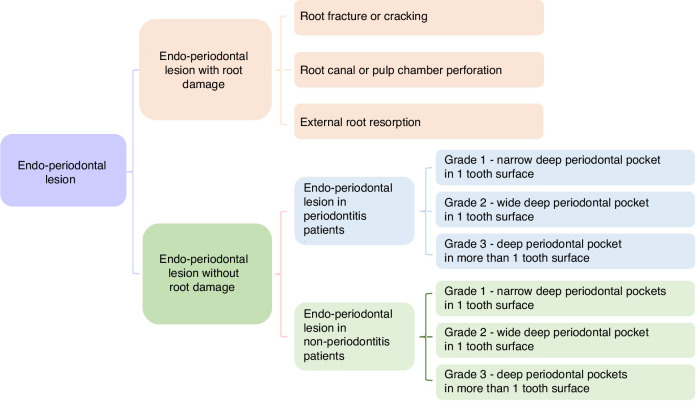
Proposal for EPL classification by the World Workshop on the Classification of Periodontal and Peri-Implant Diseases and Conditions^4^ in 2018

The classification and nomenclature system should be based on adequate assessment of symptoms and signs and should guide the therapy of the disease and determination of the prognosis. The new classification, proposed by the World Workshop on the Classification of Periodontal and Peri-Implant Diseases and Conditions in 2018, focused on the prognosis of involved tooth (Fig. 2)^4^. The classification is based primarily on the following signs and symptoms of the involved tooth: presence/absence of root damage and anatomic problems; presence/absence of full-mouth periodontitis; and severity and extent of the periodontal defect of the affected tooth, including furcation involvement.

## Clinical manifestations

EPLs can present as acute or chronic. When an EPL is caused by factors such as recent trauma, root fracture, or root perforation, an acute abscess accompanied by pain may occur^7^. However, patients who suffer periodontitis or who are receiving therapy for periodontal maintenance usually exhibit slow EPL progression with inconspicuous symptoms. In addition, once retrograde pulpitis occurs, periodontal-derived EPLs can present with acute symptoms such as spontaneous and severe pain during pulpitis^7^.

The most common clinical manifestations of EPLs include deep periodontal pockets reaching or close to the apex and an altered pulp response (including a negative response) to the pulp sensitivity test. Nearly all patients with EPLs have both clinical manifestations^7^. Other common symptoms and signs include bone resorption in the apical, lateral or furcation area of the root; spontaneous pain or percussion pain; pus discharge; tooth mobility; and sinus tract formation^7^. In addition, a small number of EPL-affected teeth present with dental crown and gingival color alterations^7^.

## Diagnosis

According to the EPL classification presented by the World Workshop on the Classification of Periodontal and Peri-Implant Diseases and Conditions in 2018, the diagnostic algorithm for EPLs is as follows.

### Step 1. Root damage detection

The main basis for judging root damage is as follows:

- Medical history: Determination of history of trauma, dental cracks, root canal therapy, periodontal mechanical therapy, orthodontic therapy, intradental bleaching, etc.
- Intraoral examination: Determination of tenderness on percussion and occlusion, periodontal condition, pulp and periapical condition, and occlusion.
- Imaging examination: This technique is an important auxiliary means to judge root injury, focusing on the presence of root fractures, root fissures, root perforations, root resorption, and root development abnormalities. Based on these results, the tooth is diagnosed as “EPL with root damage” or “EPL without root damage”.

Clinically, root dysplasia, root resorption, and root perforations may be find in the two-dimensional imaging, and can often be detected via cone-beam computed tomography (CBCT)^43,44^. However, early root fractures are difficult to diagnose. Even CBCT does not reveal 100% of root fractures^45^. Therefore, we have summarized the medical history and clinical manifestations that need to be noted in the diagnosis of root fractures as following.

- History of fractured teeth.
- Fixed-point occlusal pain in the affected tooth, meaning more pronounced occlusal pain when force is applied to a specific site.
- Occlusal abnormality.
- Narrow, deep periodontal pockets around the affected tooth.
- Sometimes the cracked root can be detected with uneven surfaces and stuck marks.
- No significant decay in the affected tooth with abnormally viable of pulp.

### Step 2. Full-mouth periodontal assessment

If the affected tooth does not have root damage, history inquiry of periodontitis and routine periodontal examination should be performed to confirm periodontitis according to the New Classification of Periodontal and Peri-Implant Diseases and Conditions in 2018. In the absence of root damage, patients with periodontitis are diagnosed as “EPL in periodontitis patients”, and patients without periodontitis are diagnosed as “EPL in non-periodontitis patients”^4,7,46^.

### Step 3. Determine the extent of periodontal destruction in the affected tooth

As long as the absence of root damage is confirmed in the affected tooth, regardless of whether the patient has periodontitis, periodontal probing and imaging examination are required to determine the extent of periodontal destruction in the EPL-affected tooth, in order to make graded diagnosis^4,7,46^.

## Factors influencing the prognosis

According to the classification system proposed in 2018, there are three main prognoses for an EPL-affected teeth: hopeless, poor, and favorable^4,7,46^. The prognosis is mainly based on the following factors: the presence or absence of root damage (the most important factor), the presence or absence of periodontitis, anatomical factors, and the extent of periodontal destruction of the involved tooth^4^. Here, we have summarized the factors affecting the prognosis of EPL patients.

### Root damage or anatomical abnormalities

EPLs with root damage associated with root fracture, pulp cavity/canal perforation, or root resorption always have unfavorable prognoses; therefore, tooth extraction is often recommended; however, sometimes there are unique cases.

1. **Root fracture:** Early detection and diagnosis of root fractures are very difficult. Even if a fracture has not been detected by CBCT, the possibility of fracture should still be condidered during subsequent periodontal surgery and apical surgery, especially for EPLs in non-periodontitis patients.
2. **Root perforations:** Extraction is generally recommended for EPL-affected teeth with root perforations. However, some root perforations can be treated. Prognosis needs to be evaluated on a case-by-case basis, and the success rate is related to the size, location, and severity of periodontal destruction, as well as the sealing capacity and biocompatibility of the restorative materials^47^.
3. **Root resorption:** Once, the size of the perforation caused by root resorption in EPL-affected teeth should be considered first because the larger the perforation is, the worse the prognosis. However, with the development of materials science, the impact of perforation, especially perforation caused by external absorption, on the prognosis gradually decreases. Second, the source of root resorption needs to be considered. Tooth retention could be achieved in cases of early internal root resorption by root canal therapy and early external root resorption by periodontal therapy^48,49^.

### Full-mouth periodontal condition of the patient

The prognosis of EPL-affected teeth is worse in patients with periodontitis than in those without periodontitis^50^. This difference may be due to the high periodontal risk and the presence of severe oral microbiota disorders in patients with periodontitis, which may also affect the efficacy of therapy^10,51^.

### Extent of periodontal destruction in EPL-affected teeth

The severity of existing periodontal destruction is an important factor in determining the prognosis of EPL-affected teeth. The prognosis is usually better in patients with narrow and deep periodontal pockets involving only one side (grade 1) than patient with periodontal destruction involving more surfaces. Experts suggest that incorporating tooth mobility as an important indicator of periodontal destruction could be an important prognostic indicator for EPL-affected teeth. However, it is worth noting that tooth mobility is affected not only by the dimensions of the alveolar bone but also by acute inflammation and occlusal trauma.

### Periodontal risk assessment of EPL-affected teeth

In addition to the severity of existing periodontal destruction in the affected tooth, the risk of future periodontal destruction is also an important factor to consider, mainly involving the following factors.

1. **Plaque control:** Plaque control is undeniably important. The impact of plaque control on the prognosis of EPL-affected teeth mainly depends on whether dental plaque is the main cause of the disease and whether good plaque control can be achieved.
  When plaque control is not the main cause of the disease, the effectiveness of improving plaque control is limited. For example, when accompanied by root resorption or fracture, although bacteria are one of the key factors in EPL progression, improving plaque control has a limited impact on prognosis.
  When plaque is the main cause of the disease and good plaque control can be achieved, the prognosis is hopeful. For example, for the EPL-affected teeth in periodontitis patients without tooth damage, the prognosis mainly depends on the control of periodontal inflammation. If good plaque control can be achieved, there is a high possibility of having a favorable long-term prognosis.
  When plaque is the main cause of disease but good plaque control is difficult to achieve, the long-term prognosis depends on the specific situation. The reasons for poor plaque control may vary; for example, patient compliance may be improved through repeated oral health education, and the prognosis may be favorable. However, when the factors that affect plaque control are difficult to change, it is very difficult to achieve a favorable long-term prognosis. For example, some anatomical limitations, such as furcation involvement between the buccal and palatal roots in maxillary molars, can hinder the implementation of effective plaque control and lead to a poor prognosis for EPL-involved teeth^50^.
2. **Occlusal burden:** A history of uncontrollable occlusal trauma to the affected tooth significantly increases the risk of periodontal destruction of the affected tooth, leading to a poor prognosis^52^.
3. **General condition:** The patient’s general condition is also an important factor affecting whether an EPL-affected tooth with EPL can be saved. When the prognosis of EPL-affected teeth is uncertain, treatment decisions tend to favor the extraction of teeth from patients with poor glycemic control^53^, heavy smokers^54^, or those under mental stress^55^.

### Difficulty of root canal therapy for EPL-affected teeth

The prognosis of EPL-affected teeth is also related to the periapical index and the root canal system^56^. The better the apical closure and the simpler the root canal system, the better the outcome of root canal therapy in EPL-affected teeth is. The difficulty of root canal therapy worsens the prognosis of teeth affected by EPLs^56^.

### Patients’ dentition condition and subsequent prosthodontic treatment plan

For teeth with an uncertain prognosis, intact dentition is more conducive to tooth preservation. For the patients with pre-existing dentition defects, it is necessary to decide whether to preserve the tooth based on the subsequent restoration plan. Multi-root tooth with deep periodontal pockets, severe tooth mobility and furcation involvement are recommended for extraction, which is beneficial for complex restorative therapy^47^.

### Sociopsychological factors

Sociopsychological factors include the patient’s financial status, psychological expectations about the course of therapy and prognosis. In cases of teeth with uncertain prognoses, the patient’s preference is crucial^57^. The treatment of EPLs is complex and often costly. Therefore, patients should be fully informed of the condition of the affected tooth, necessary medical investments (time, money, and discomfort during therapy), medical risks, and therapy options. Additionally, the patient’s economic status and psychological expectations should be fully considered^57^. Reasonable therapy decisions should be made under the premise of adequate and effective communication between doctors and patients.

## Treatment decision-making and therapy procedures

EPLs encompass a spectrum of diseases characterized by diverse etiologies and varying severity, leading to a wide range of prognoses. Therapeutic decision-making and treatment procedures are key factors for the successful treatment of EPL-affected teeth. Therefore, this section elaborates on both separately.

### Treatment decision-making for EPLs

**Fig. 3:**
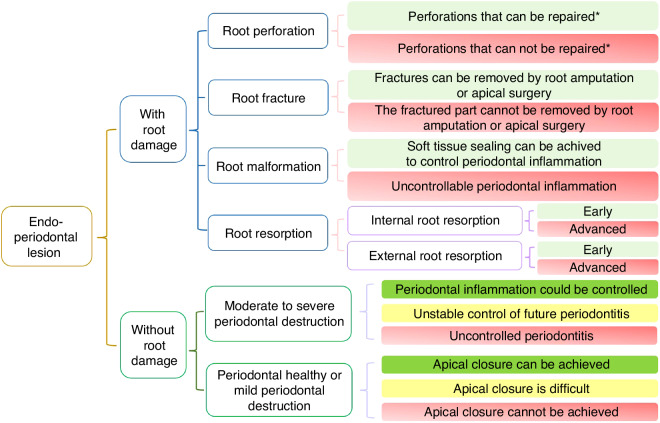
Decision-making chart for the EPLs treatment^1,47–49,58–64^.  Tooth extraction is recommended.  Inconclusive, usually depends on the patient's willingness.  Attempt to treat the tooth.  Tooth conservation is recommended. * Whether root perforations can be repaired depends not only on the injury itself but also on the clinician's skills, experience, and other factors

To concisely illustrate the treatment decision-making process for EPL-involved teeth, the expert group drew a decision-making tree for EPL therapy (Fig. 3)^1,47–49,58–64^. Note that the decision-making tree considers the condition of the EPL-affected tooth itself, rather than the patient’s dentition, subsequent restoration plan, and sociopsychological factors, which mainly provide treatment decision preferences for teeth with an uncertain prognosis.

### Therapy procedures

According to the treatment decision tree for teeth affected by EPLs (Fig. 3), with the exception of teeth that must be extracted, most EPL-affected teeth should first undergo endodontic therapy whether root damage is present or absent. It is advisable to allow sufficient time for re-evaluation before deciding whether to proceed with periodontal surgery^65–67^.

Patients with periodontitis and EPL-affected teeth without root damage are usually diagnosed with stage III or IV periodontitis. Treating EPL-affected teeth is part of a comprehensive periodontal therapy, with the goal of preserving natural teeth and maintaining complete dentition^68,69^. If pulp disease secondary to periodontitis occurs and periodontal inflammation is controllable in the future, it is recommended to treat acute symptoms first and control pulp infection. Nonsurgical periodontal therapy can be performed concurrently with endodontic therapy^70^, while periodontal surgical therapy should be carried out after completing endodontic therapy. Periodontal treatment is recommended according to the S3 level clinical practice guidelines for periodontitis treatment by the European Federation of Periodontology (EFP)^68,69,71^.

## Therapy techniques

Accurate prognostic assessment is the cornerstone of the therapeutic decision-making process for EPLs. It is imperative to initially conduct a thorough prognostic evaluation before embarking on tailored therapy. In the management of EPL-affected teeth, it is crucial to employ an integrated approach that combines both endodontic and periodontal therapies. The subsequent content outlines therapy to save teeth with EPLs.

### Root canal treatment

The prevailing consensus recommends the use of endodontic intervention prior to periodontal procedures because root canal therapy may have the following potential benefits. Perfect root canal therapy can effectively eliminate the transportation of infectious substances, control pulp infection, reduce pain and other discomfort, and promote periodontal tissue healing. Moreover, root canal treatment, by clearing infectious material and filling and sealing the root canals, prevents further internal resorption in EPL-affected teeth with internal root resorption, thus reducing the risk of root fractures or the need for extraction. The thoroughness of root canal therapy is a critical determinant of the prognosis of EPL-affected teeth^56^.

Clinical evidence from a randomized controlled trial demonstrated the efficacy of Ca(OH)_2_ and a 2% chlorhexidine + Ca(OH)_2_ intracanal medicament formulation. Relative to a control cohort that was not treated with these pharmacological agents, a marked decrease in pocket depth and bleeding on probing, coupled with increased attachment levels, was observed across all test groups within a six-month period. Despite the absence of direct corroborative evidence linking intracanal medicaments to enhanced periodontal healing, these findings underscore the important role of removing infected pulp tissue in facilitating periodontal recovery^72^. Raheja et al. also proved that chlorhexidine, when used as an effective intracanal medication, can promote periodontal healing after EPL-related periodontal surgical therapy^73^.

### Steps 1 and 2. Periodontal therapy procedures

For EPL-affected teeth without root damage and with severe periodontal destruction, the clinical outcome largely depends on the effectiveness of periodontal therapy. Moreover, the long-term control of periodontal disease and the sustainability of the affected teeth are primarily dependent on consistent and thorough maintenance therapy. In the context of EPLs, the initial stage of periodontal therapy, encompassing step 1 “Controlling Risk Factors” and step 2 “Etiological Therapy,” is an indispensable component of the therapeutic strategy^71^. Controlling risk factors involves oral hygiene instruction, supragingival scaling, and the management of systemic risk factors such as diabetes, aiming to achieve good plaque self- control, remove supragingival plaque and calculus, and control the progression of periodontal disease. Etiological therapy consists of removing subgingival plaque and calculus via subgingival scaling and root planning, as well as ancillary therapy methods. Following the healing interval, the individual’s reaction to the step 2 intervention is assessed via complete periodontal reevaluation. If there are still presence of pockets ≥4 mm with bleeding on probing or presence of deep periodontal pockets ≥6 mm after step 2 of the intervention, step 3 of the protocol, the surgical therapy, should be initiated. If these therapeutic goals are fulfilled, it is recommended that the patient participate in an organized periodontal supportive care program.

### Surgical therapy

For EPL-affected teeth without root damage and periodontitis diagnosed as “Endo-periodontal lesion in non-periodontitis patients” in the New Classification of Periodontal and Peri-Implant Diseases and Conditions in 2018 (usually corresponding to “primary endodontic lesions with secondary periodontal involvement” or “primary endodontic lesions” in the 1972 classification), root canal treatment with or without non-surgical periodontal therapy can restore health. However, some EPL-affected teeth require additional surgical treatment beyond root canal treatment and non-surgical periodontal treatment. Surgery can still be performed to save EPL-affected teeth with limited root damage. For EPL-affected teeth without root damage, the prognosis mainly depends on the severity and extent of periodontal destruction. When periodontal damage is extensive and severe, surgery should be recommended with caution. Surgical treatment methods commonly used for EPL-affected teeth are described here.

1. **Root amputation or hemisection**: This procedure involves the removal of one or two of the most severely damaged roots of a multirooted tooth while retaining the crown and the remaining roots to continue functioning. In EPL-affected teeth, root amputation is often used when one or two roots of a multirooted tooth have severe periodontal tissue damage or when a molar root has suffered damage, such as a vertical fracture, horizontal fracture, or perforation, while the other roots are intact and can undergo thorough root canal treatment. Root amputation requires prior root canal treatment, thus potentially necessitating more complex therapy and higher economic and time costs. However, as a method that allows for tooth preservation and delayed implant therapy, it is a worthwhile option^74^.
  Currently, with the development of dental implant technology, hemisection is less commonly used because there is still a need to repair “the missing half of the tooth” after hemisection. The possible indication for hemisection for EPL-involved tooth may be: (1)For issues related to the distal root of lower second molar, the distal part needs to be removed; (2)The remaining “tooth” after hemisection surgery can be used as the abutment tooth for the “missing tooth” along with the adjacent tooth that require crown restoration.
2. **Endodontic surgery**: The endodontic surgical procedures commonly employed for EPLs include apical resection and root surface repair. When the tooth fracture is limited to the apical part of the root, apical resection can be considered to preserve the affected tooth. In cases of EPL-affected teeth with perforations or developmental anomalies of the root surface, root surface repair surgeries are also recommended^75^.
  Studies have shown that in maxillary lateral incisors with deep palatal grooves accompanied by pulp necrosis and extensive periodontal pockets, teeth can be preserved through a combination of root canal treatment, endodontic surgery, and periodontal regenerative surgery using enamel matrix derivative proteins^76^.
  Regardless of the type of endodontic surgery performed, adequate root sealing, which requires good sealing biomaterials, is critical. Among all the biomaterials utilized in endodontic surgery, the evidence on the effectiveness of mineral trioxide aggregate (MTA) is the most comprehensive, and iRoot BP Plus has also begun to be widely used in clinical practice^77^.
3. **Periodontal flap surgery:** If preservation of EPL-affected teeth in patients with periodontitis is planned (most of the time corresponding to “primary periodontal lesions with secondary endodontic involvement” and “primary periodontal lesions” according to the 1972 classification), periodontal flap surgery is the most commonly recommended surgery. A prospective randomized clinical trial by Tewari revealed that flap surgery combined with root canal therapy effectively healed periodontal pockets in EPLs with root apex communication, reducing the probing depth from an average of 12.16–4.94 mm^78^.
4. **Regenerative periodontal surgery**: Whether it is root amputation, hemisection, endodontic surgery or periodontal flap surgery, it is possible to consider using regenerative materials to promote tissue healing during the surgery. A systematic review incorporating three randomized controlled trials and a prospective single-arm study revealed that following regenerative surgery on 125 EPL-affected teeth^79^, 58.4% of teeth achieved complete healing, 24% exhibited scar tissue formation/incomplete healing, and 12.8% had uncertain healing outcomes, while 4.8% exhibited therapy failure. This may be attributed to the complexity of EPLs as a disease and the sensitivity of the GTR technique. For the EPL-affected tooth treated by GTR surgery, if the depth of periodontal pockets remains deeper than 6 mm after sufficient observation time post-surgery, GTR can be repeated to reach the periodontal therapy endpoints^79^. Given that some regenerative materials are derived from pigs or cows, attention should be given to potential religious concerns among certain populations^69^.
5. **Intentional replantation surgery for EPLs:** Initially reported by Counsell, intentional replantation surgery is a potential therapy for EPLs. This approach is considered for conditions such as root canal wall perforations, teeth with significant developmental anomalies, or in cases where a patient strongly desires to preserve a tooth despite a longitudinal root fracture^80,81^. The procedure involves extracting the affected tooth in its entirety, conducting root canal treatment, filling extracorporeally, removing infected tissues, severing sources of infection, and finally replanting the treated tooth back into its original tooth socket. This surgical technique aims to eliminate the causes of pulp-periapical and periodontal diseases, allowing the periodontal tissues to be repaired, and maintaining masticatory function. The success rate of this surgery is strongly dependent on the dentist’s experience and expertise. The dentist must complete the entire surgical process within a short duration, ensuring complete tooth extraction, thorough cleaning of extraction socket and root, resection of 2–3 mm of root apex to seal apical foramen, and striving to preserve periodontal ligament. Additionally, the duration of surgery is crucial. It is typically completed within 15 minutes to minimize the risk of infection. Saida, in a prospective two-year follow-up study of cases, found that intentional replantation might be a viable therapy for EPLs and for teeth deemed otherwise untreatable^82^. For the tooth with deep palatal groove, intentional replantation after root canal treatment also shows promise for tooth preservation^80,81,83^.

The above treatment targetes EPL-affected teeth that are intended to be saved. For these affected teeth, root canal treatment is usually recommended first, followed by periodontal non-surgical therapy. Periodontal surgery, such as root amputation and hemisection, can be considered if necessary. If the above treatments fail, the affected tooth still needs to be extracted.

## Prognosis

After discussion, the expert group developed the following criteria for successful treatment of EPLs include: (1) conscious improvement of symptoms and normal function of the affected tooth; (2) no recurrent abscess or fistula; (3) control of periodontal inflammation through the elimination of deep periodontal pockets, and the improvement of the height of alveolar bone to a certain extent; and (4) control of pulp and periapical inflammation, which is exemplified by significant improvement in periapical bone destruction on postoperative follow-up imaging.

A longer follow-up period is typically better. Generally, significant imaging changes can be observed within 3 months after treatment. Therefore, it seems reasonable to recommend a follow-up period of at least 3 months. However, persistent residual infection should also be considered. Therefore, some scholars believe that a minimum observation period of 4 years is necessary^77^.

The prognosis of EPLs depends on their diagnosis and treatment, and the reported survival rates vary greatly; randomized controlled trials are rare in the literature. Overall, EPL-affected teeth with root damage usually have a poor prognosis; for teeth without root damage, the prognosis mainly depends on the severity of periodontal destruction. Therefore, the new 2018 classification system for the prognosis of EPLs can aid in accurately estimating patient prognosis. However, it is worth noting that although the new classification system based on prognosis has advantages in prognosis determination, etiological analysis is also a very important part of prognosis judgment, and etiological treatment is still important for improving prognosis. For example, for an EPL-involved tooth with a root fracture caused by occlusal trauma, successful root amputation is only one required element for a good prognosis; occlusal balance is also an important factor affecting the long-term prognosis of affected teeth.

## Conclusions

EPLs intricately involve both periodontal and pulpal tissues. The intricate interplay of infection or inflammation through both physiological and pathological channels, such as the apical foramen and root damage, adds complexity to the condition. A precise and timely diagnosis is paramount for guiding effective therapy and improving the prognosis. Early identification of the underlying causes of EPLs is essential for formulating a therapy strategy tailored to specific classifications, involving carefully sequenced and varied therapy approaches. It is imperative for dentists to embrace the philosophy of integrated periodontal and endodontic therapy, fostering interdisciplinary collaboration for a holistic and systematic approach in diagnosis and therapy. Such concerted efforts by periodontists and endodontists will lead to a unified understanding and management of EPLs, with the ultimate goal of maximally prolonging the lifespan of the affected tooth.
